# Supplementary material for: Prevalence of non-communicable diseases among individuals with HIV infection by antiretroviral therapy status in Dar es Salaam, Tanzania
Source: PLoS One. 2020 Jul 9;15(7):e0235542. doi: 10.1371/journal.pone.0235542 (PMC7347196; doi:10.1371/journal.pone.0235542)
Supplement: S1 Table — (DOCX) [file pone.0235542.s001.docx]

**S1 Table. Comparison of socio-demographic and clinical characteristics of people with HIV infection in 3 ART status groups.**

| **Characteristic** | **Total**  **N = 612** | **ART status** | | | **p-value** |
| --- | --- | --- | --- | --- | --- |
|  |  | **Naïve**  **306 (50.0 %)** | **5-10 years**  **183 (29.9 %)** | **>10 years**  **123 (20.1 %)** |  |
| **Mean age ± SD (years)** | 41.2 ± 12.8 | 37.5 ± 11.8 | 43.7 ± 12.5 | 46.7 ± 12.8 | <0.001 |
|  |  |  |  |  |  |
| **Age groups (years)** |  |  |  |  |  |
| <40 | 273 (44.6%) | 196 (64.1%) | 54 (29.5%) | 23 (18.7%) |  |
| ≥40 | 339 (55.4%) | 110 (35.9%) | 129 (70.5%) | 100 (81.3%) | <0.001 |
| **Sex** |  |  |  |  |  |
| Male | 184 (30.1%) | 98 (32.0%) | 48 (26.2%) | 38 (30.9%) |  |
| Female | 428 (69.9%) | 208 (68.0%) | 135 (73.8%) | 85 (69.1%) | 0.391 |
| **Marital status** |  |  |  |  |  |
| Single | 174 (28.4%) | 98 (32.0%) | 46 (25.1%) | 30 (24.4%) |  |
| Ever married | 438 (71.6%) | 208 (68.0%) | 137 (74.9%) | 93 (75.6%) | 0.142 |
| **Education level** |  |  |  |  |  |
| None | 46 (7.5%) | 33 (10.8%) | 10 (5.5%) | 3 (2.4%) |  |
| Primary school | 358 (58.5%) | 203 (66.3%) | 96 (52.5%) | 59 (48.0%) |  |
| Above primary school | 208 (34.0%) | 70 (22.9%) | 77 (42.1%) | 61 (49.6%) | <0.001 |
| **Occupation** |  |  |  |  |  |
| Not employed | 171 (27.9%) | 79 (25.8%) | 54 (29.5%) | 38 (30.9%) |  |
| Employed | 115 (18.8%) | 37 (12.1%) | 44 (24.0%) | 34 (27.6%) | <0.001 |
| Self employed | 326 (53.3%) | 190 (62.1%) | 85 (46.4%) | 51 (41.5%) |  |
| **Smoking** |  |  |  |  |  |
| No | 583 (95.3%) | 287 (93.8%) | 175 (95.6%) | 121 (98.4%) |  |
| Yes | 29 (4.7%) | 19 (6.2%) | 8 (4.4%) | 2 (1.6%) | 0.125 |
| **Alcohol** |  |  |  |  |  |
| No | 442 (72.2%) | 237 (77.5%) | 126 (68.9%) | 79 (64.2%) |  |
| Yes | 170 (27.8%) | 69 (22.5%) | 57 (31.1%) | 44 (35.8%) | 0.010 |
| **Level of physical activity** |  |  |  |  |  |
| Moderate /vigorous intensity | 83 (13.6%) | 18 (5.9%) | 31 (16.9%) | 34 (27.6%) |  |
| Low intensity | 529 (86.4%) | 288 (94.1%) | 152 (83.1%) | 89 (72.4%) | <0.001 |
| **BMI** |  |  |  |  |  |
| Underweight/Normal | 350 (57.2%) | 219 (71.6%) | 82 (44.8%) | 49 (39.8%) |  |
| Overweight/obesity | 262 (42.8%) | 87 (28.4%) | 101 (55.2%) | 74 (60.2%) | <0.001 |
| **Family history of HTN** |  |  |  |  |  |
| No | 552 (90.2%) | 272 (88.9%) | 170 (92.9%) | 110 (89.4%) |  |
| Yes | 60 (9.8%) | 34 (11.1%) | 13 (7.1%) | 13 (10.6%) | 0.336 |
| **Family history of DM** |  |  |  |  |  |
| No | 571 (93.3%) | 285 (93.1%) | 172 (94.0%) | 114 (92.7%) |  |
| Yes | 41 (6.7%) | 21 (6.9%) | 11 (6.0%) | 9 (7.3%) | 0.893 |

ART: antiretroviral therapy; BMI: body mass index; DM: diabetes mellitus; HTN: hypertension.
