# Supplementary material for: Prevalence of non-communicable diseases among individuals with HIV infection by antiretroviral therapy status in Dar es Salaam, Tanzania
Source: PLoS One. 2020 Jul 9;15(7):e0235542. doi: 10.1371/journal.pone.0235542 (PMC7347196; doi:10.1371/journal.pone.0235542)
Supplement: S2 Table — (DOCX) [file pone.0235542.s002.docx]

**S2 Table. Clinical and laboratory findings among study subjects in 3 ART status groups.**

| **Variable** | **Total**  **N=612** | **ART status** | | | **p-value** |
| --- | --- | --- | --- | --- | --- |
|  |  | **ART naive** | **ART 5-10 years** | **ART >10 years** |  |
| **Hypertension** |  |  |  |  |  |
| Yes | 98 (16.0%) | 21 (6.9%) | 41 (22.4%) | 36 (29.3%) | <0.001 |
| No | 514 (84.0%) | 285 (93.1%) | 142 (77.6%) | 87 (70.7%) |  |
| **Impaired glucose tolerance** |  |  |  |  |  |
| Yes | 84 (13.7%) | 14 (4.6%) | 39 (21.3%) | 31 (25.2%) | <0.001 |
| Other | 528 (86.3%) | 292 (95.4%) | 144 (78.7%) | 92 (74.8%) |  |
| **Diabetes mellitus** |  |  |  |  |  |
| Yes | 64 (10.5%) | 12 (3.9%) | 26 (14.2%) | 26 (21.1%) | <0.001 |
| No | 548 (89.5%) | 294 (96.1%) | 157 (85.8%) | 97 (78.9%) |  |
| **Renal dysfunction** |  |  |  |  |  |
| Yes | 30 (4.9%) | 17 (5.6%) | 7 (3.8%) | 6 (4.9%) | 0.692 |
| No | 582 (95.1%) | 289 (94.4%) | 176 (96.2%) | 117 (95.1%) |  |
| **Hypercholesterolemia** |  |  |  |  |  |
| Yes | 144 (23.5%) | 51 (16.7%) | 53 (29.0%) | 40 (32.5%) | <0.001 |
| No | 468 (76.5%) | 255 (83.3%) | 130 (71.0%) | 83 (67.5%) |  |
| **Hypertriglyceridemia** |  |  |  |  |  |
| Yes | 78 (12.7%) | 29 (9.5%) | 24 (13.1%) | 25 (20.3%) | 0.009 |
| No | 534 (87.3%) | 277 (90.5%) | 159 (86.9%) | 98 (79.7%) |  |
| **Low HDL cholesterol** |  |  |  |  |  |
| Yes | 158 (25.8%) | 87 (28.4%) | 44 (24.0%) | 27 (22.0%) | 0.308 |
| No | 454 (74.2%) | 219 (71.6%) | 139 (76.0%) | 96 (78.0%) |  |
| **High LDL cholesterol** |  |  |  |  |  |
| Yes | 157 (25.7%) | 87 (28.4%) | 44 (24.0%) | 26 (21.0%) | 0.246 |
| No | 455 (74.3%) | 219 (71.6%) | 139 (76.0%) | 97 (78.9%) |  |

ART: antiretroviral therapy; HDL: high density lipoprotein; LDL: low density lipoprotein.
