# Supplementary material for: Prevalence of non-communicable diseases among individuals with HIV infection by antiretroviral therapy status in Dar es Salaam, Tanzania
Source: PLoS One. 2020 Jul 9;15(7):e0235542. doi: 10.1371/journal.pone.0235542 (PMC7347196; doi:10.1371/journal.pone.0235542)
Supplement: S3 Table — (DOCX) [file pone.0235542.s003.docx]

**S3A Table. Factors associated with hypertension among individuals with HIV infection.**

| **Variable** | **Total**  **N=612** | **Hypertension**  **n=98** | **PR (95% CI)** | **p-value** | **Adjusted PR**  **(95% CI)** | **p-value** |
| --- | --- | --- | --- | --- | --- | --- |
| **ART status** |  |  |  |  |  |  |
| ≥5 years | 306 | 77 (25.2%) | 3.67 (2.32-5.79) | <0.001 | 1.94 (1.20-3.14) | 0.007 |
| Naïve | 306 | 21 (6.9%) | 1 |  | 1 |  |
| **Age** | - | - | 1.06 (1.05-1.07) | <0.001 | 1.06 (1.04-1.07) | <0.001 |
| **Sex** |  |  |  |  |  |  |
| Male | 184 | 37 (20.1%) | 1 |  | 1 |  |
| Female | 428 | 61 (14.3%) | 0.71 (0.49-1.03) | 0.069 | 0.94 (0.65-1.35) | 0.723 |
| **Smoking status** |  |  |  |  |  |  |
| Yes | 29 | 2 (6.9%) | 0.42 (0.11-1.62) | 0.207 | - | - |
| No | 583 | 96 (16.5%) | 1 |  |  |  |
| **Alcohol use** |  |  |  |  |  |  |
| Yes | 170 | 29 (17.1%) | 1.09 (0.74-1.62) | 0.661 | - | - |
| No | 442 | 69 (15.6%) | 1 |  |  |  |
| **Physical activity** |  |  |  |  |  |  |
| Moderate/Vigorous | 83 | 19 (22.9%) | 1.53 (0.98-2.39) | 0.060 | 1.63 (1.03-2.58) | 0.035 |
| Low intensity | 529 | 79 (14.9%) | 1 |  | 1 |  |
| **BMI** | - | - | 1.07 (1.05-1.09) | <0.001 | 1.05 (1.02-1.07) | <0.001 |

ART: antiretroviral therapy; BMI: body mass index; CI: confidence interval; PR: prevalence ratio.

**S3B Table. Factors associated with impaired glucose tolerance among individuals with HIV infection.**

| **Variable** | **Total**  **N=612** | **IGT**  **n=84** | **PR (95% CI)** | **p-value** | **Adjusted PR**  **(95% CI)** | **p-value** |
| --- | --- | --- | --- | --- | --- | --- |
| **ART status** |  |  |  |  |  |  |
| ≥5 years | 306 | 70 (22.9%) | 5.00 (2.88-8.68) | <0.001 | 2.49 (1.41-4.42) | 0.002 |
| Naïve | 306 | 14 (4.6%) | 1 |  | 1 |  |
| **Age** | - | - | 1.06 (1.05-1.07) | <0.001 | 1.06 (1.04-1.07) | <0.001 |
| **Sex** |  |  |  |  |  |  |
| Male | 184 | 27 (14.7%) | 1 |  |  |  |
| Female | 428 | 57 (13.3%) | 0.91 (0.59-1.39) | 0.654 | - | - |
| **Smoking status** |  |  |  |  |  |  |
| Yes | 29 | 1 (3.4%) | 0.24 (0.03-1.68) | 0.152 | 0.29 (0.04-1.93) | 0.199 |
| No | 583 | 83 (14.2%) | 1 |  | 1 |  |
| **Alcohol use** |  |  |  |  |  |  |
| Yes | 170 | 28 (16.5%) | 1.30 (0.86-1.97) | 0.219 | - | - |
| No | 442 | 56 (12.7%) | 1 |  |  |  |
| **Physical activity** |  |  |  |  |  |  |
| Moderate/Vigorous | 83 | 15 (18.1%) | 1.39 (0.83-2.30) | 0.209 | - | - |
| Low intensity | 529 | 69 (13.0%) | 1 |  |  |  |
| **BMI** | - | - | 1.09 (1.07-1.11) | <0.001 | 1.07 (1.04-1.09) | <0.001 |

ART: antiretroviral therapy; BMI: body mass index; CI: confidence interval; IGT: impaired glucose tolerance; PR: prevalence ratio.

**S3C Table. Factors associated with diabetes mellitus among individuals with HIV infection.**

| **Variable** | **Total**  **N=612** | **DM**  **n=64** | **PR (95% CI)** | **p-value** | **Adjusted PR**  **(95% CI)** | **p-value** |
| --- | --- | --- | --- | --- | --- | --- |
| **ART status** |  |  |  |  |  |  |
| ≥5 years | 306 | 52 (17.0%) | 4.33 (2.36-7.96) | <0.001 | 2.17 (1.15-4.10) | 0.017 |
| Naïve | 306 | 12 (3.9%) | 1 |  | 1 |  |
| **Age** | - | - | 1.06 (1.04-1.07) | <0.001 | 1.05 (1.03-1.07) | <0.001 |
| **Sex** |  |  |  |  |  |  |
| Male | 184 | 20 (10.9%) | 1 |  |  |  |
| Female | 428 | 44 (10.3%) | 0.95 (0.57-1.56) | 0.827 | - | - |
| **Smoking status** |  |  |  |  |  |  |
| Yes | 29 | 1 (3.4%) | 0.31 (0.05-2.22) | 0.249 | - | - |
| No | 583 | 63 (10.8%) | 1 |  |  |  |
| **Alcohol use** |  |  |  |  |  |  |
| Yes | 170 | 22 (12.9%) | 1.36 (0.84-2.21) | 0.212 | - | - |
| No | 442 | 42 (9.5%) | 1 |  |  |  |
| **Physical activity** |  |  |  |  |  |  |
| Moderate/Vigorous | 83 | 6 (7.2%) | 0.66 (0.29-1.48) | 0.313 | - | - |
| Low intensity | 529 | 58 (11.0%) | 1 |  |  |  |
| **BMI** | - | - | 1.10 (1.07-1.13) | <0.001 | 1.08 (1.05-1.11) | <0.001 |

ART: antiretroviral therapy; BMI: body mass index; CI: confidence interval; DM: diabetes mellitus; PR: prevalence ratio.

**S3D Table. Factors associated with renal dysfunction among individuals with HIV infection.**

| **Variable** | **Total**  **N=612** | **Renal dysfunction**  **n=30** | **PR (95% CI)** | **p-value** | **Adjusted PR**  **(95% CI)** | **p-value** |
| --- | --- | --- | --- | --- | --- | --- |
| **ART status** |  |  |  |  |  |  |
| ≥5 years | 306 | 13 (4.2%) | 0.76 (0.38-1.55) | 0.456 | - | - |
| Naïve | 306 | 17 (5.6%) | 1 |  |  |  |
| **Age** | - | - | 1.05 (1.02-1.08) | <0.001 | - | - |
| **Sex** |  |  |  |  |  |  |
| Male | 184 | 11 (6.0%) | 1 |  |  |  |
| Female | 428 | 19 (4.4%) | 0.74 (0.36-1.53) | 0.420 | - | - |
| **Smoking status** |  |  |  |  |  |  |
| Yes | 29 | 1 (3.4%) | 0.69 (0.10-4.92) | 0.714 | - | - |
| No | 583 | 29 (5.0%) | 1 |  |  |  |
| **Alcohol use** |  |  |  |  |  |  |
| Yes | 170 | 8 (4.7%) | 0.95 (0.43-2.08) | 0.889 | - | - |
| No | 442 | 22 (5.0%) | 1 |  |  |  |
| **Physical activity** |  |  |  |  |  |  |
| Moderate/Vigorous | 83 | 4 (4.8%) | 0.98 (0.35-2.74) | 0.970 | - | - |
| Low intensity | 529 | 26 (4.9%) | 1 |  |  |  |
| **BMI** | - | - | 1.01 (0.95-1.06) | 0.854 | - | - |

ART: antiretroviral therapy; BMI: body mass index; CI: confidence interval; PR: prevalence ratio.

**S3E Table. Factors associated with hypercholesterolemia among individuals with HIV infection.**

| **Variable** | **Total**  **N=612** | **Hypercholesterolemia**  **n=144** | **PR (95% CI)** | **p-value** | **Adjusted PR**  **(95% CI)** | **p-value** |
| --- | --- | --- | --- | --- | --- | --- |
| **ART status** |  |  |  |  |  |  |
| ≥5 years | 306 | 93 (30.4%) | 1.82 (1.35-2.47) | <0.001 | 1.33 (0.96-1.84) | 0.085 |
| Naïve | 306 | 51 (16.7%) | 1 |  | 1 |  |
| **Age** | - | - | 1.02 (1.01-1.03) | <0.001 | 1.01 (1.00-1.03) | 0.013 |
| **Sex** |  |  |  |  |  |  |
| Male | 184 | 32 (17.4%) | 1 |  |  |  |
| Female | 428 | 112 (26.2%) | 1.50 (1.06-2.14) | 0.023 | 1.40 (0.97-2.02) | 0.073 |
| **Smoking status** |  |  |  |  |  |  |
| Yes | 29 | 2 (6.9%) | 0.28 (0.07-1.09) | 0.066 | 0.36 (0.10-1.30) | 0.118 |
| No | 583 | 142 (24.4%) | 1 |  |  |  |
| **Alcohol use** |  |  |  |  |  |  |
| Yes | 170 | 41 (24.1%) | 1.03 (0.75-1.42) | 0.831 | - | - |
| No | 442 | 103 (23.3%) | 1 |  |  |  |
| **Physical activity** |  |  |  |  |  |  |
| Moderate/Vigorous | 83 | 13 (15.7%) | 0.63 (0.38-1.07) | 0.085 | 0.65 (0.39-1.09) | 0.101 |
| Low intensity | 529 | 131 (24.8%) | 1 |  |  |  |
| **BMI** | - | - | 1.06 (1.04-1.08) | <0.001 | 1.05 (1.03-1.07) | <0.001 |

ART: antiretroviral therapy; BMI: body mass index; CI: confidence interval; PR: prevalence ratio.

**S3F Table. Factors associated with hypertriglyceridemia among individuals with HIV infection.**

| **Variable** | **Total**  **N=612** | **Hypertriglyceridemia**  **n=78** | **PR (95% CI)** | **p-value** | **Adjusted PR**  **(95% CI)** | **p-value** |
| --- | --- | --- | --- | --- | --- | --- |
| **ART status** |  |  |  |  |  |  |
| ≥5 years | 306 | 49 (16.0%) | 1.69 (1.10-2.60) | 0.017 | 1.40 (0.90-2.17) | 0.131 |
| Naïve | 306 | 29 (9.5%) | 1 |  | 1 |  |
| **Age** | - | - | 1.03 (1.01-1.04) | <0.001 | 1.02 (1.00-1.03) | 0.017 |
| **Sex** |  |  |  |  |  |  |
| Male | 184 | 34 (18.5%) | 1 |  |  |  |
| Female | 428 | 44 (10.3%) | 0.56 (0.37-0.84) | 0.005 | 0.59 (0.39-0.90) | 0.015 |
| **Smoking status** |  |  |  |  |  |  |
| Yes | 29 | 3 (10.3%) | 0.80 (0.27-2.40) | 0.696 | - | - |
| No | 583 | 75 (12.9%) | 1 |  |  |  |
| **Alcohol use** |  |  |  |  |  |  |
| Yes | 170 | 25 (14.7%) | 1.23 (0.79-1.91) | 0.365 | - | - |
| No | 442 | 53 (12.0%) | 1 |  |  |  |
| **Physical activity** |  |  |  |  |  |  |
| Moderate/Vigorous | 83 | 11 (13.3%) | 1.05 (0.58-1.90) | 0.881 | - | - |
| Low intensity | 529 | 67 (12.7%) | 1 |  |  |  |
| **BMI** | - | - | 1.04 (1.00-1.07) | 0.027 | 1.02 (0.99-1.06) | 0.248 |

ART: antiretroviral therapy; BMI: body mass index; CI: confidence interval; PR: prevalence ratio.

**S3G Table. Factors associated with low HDL cholesterol among individuals with HIV infection.**

| **Variable** | **Total**  **N=612** | **Low HDL cholesterol**  **n=158** | **PR (95% CI)** | **p-value** | **Adjusted PR**  **(95% CI)** | **p-value** |
| --- | --- | --- | --- | --- | --- | --- |
| **ART status** |  |  |  |  |  |  |
| ≥5 years | 306 | 71 (23.2%) | 0.82 (0.62-1.07) | 0.141 | 0.75 (0.55-1.01) | 0.057 |
| Naïve | 306 | 87 (28.4%) | 1 |  | 1 |  |
| **Age** | - | - | 0.99 (0.98-1.00) | 0.025 | 0.99 (0.98-1.00) | 0.071 |
| **Sex** |  |  |  |  |  |  |
| Male | 184 | 34 (18.5%) | 1 |  | 1 |  |
| Female | 428 | 124 (29.0%) | 1.57 (1.12-2.20) | 0.009 | 1.48 (1.04-2.10) | 0.029 |
| **Smoking status** |  |  |  |  |  |  |
| Yes | 29 | 4 (13.8%) | 0.52 (0.21-1.31) | 0.167 | 0.66 (0.26-1.66) | 0.375 |
| No | 583 | 154 (26.4%) | 1 |  | 1 |  |
| **Alcohol use** |  |  |  |  |  |  |
| Yes | 170 | 38 (22.4%) | 0.82 (0.60-1.13) | 0.233 | - | - |
| No | 442 | 120 (27.1%) | 1 |  |  |  |
| **Physical activity** |  |  |  |  |  |  |
| Moderate/Vigorous | 83 | 18 (21.7%) | 0.82 (0.53-1.26) | 0.368 | - | - |
| Low intensity | 529 | 140 (26.5%) | 1 |  |  |  |
| **BMI** | - | - | 1.02 (1.00-1.04) | 0.041 | 1.03 (1.02-1.05) | <0.001 |

ART: antiretroviral therapy; BMI: body mass index; CI: confidence interval; HDL: High density lipoprotein; PR: prevalence ratio.

**S3H Table. Factors associated with high LDL cholesterol among individuals with HIV infection.**

| **Variable** | **Total**  **N=612** | **High LDL cholesterol**  **n=157** | **PR (95% CI)** | **p-value** | **Adjusted PR**  **(95% CI)** | **p-value** |
| --- | --- | --- | --- | --- | --- | --- |
| **ART status** |  |  |  |  |  |  |
| ≥5 years | 306 | 70 (22.9%) | 0.80 (0.61-1.06) | 0.117 | 0.75 (0.55-1.02) | 0.066 |
| Naïve | 306 | 87 (28.4%) | 1 |  | 1 |  |
| **Age** | - | - | 0.99 (0.98-1.00) | 0.157 | 0.99 (0.98-1.01) | 0.311 |
| **Sex** |  |  |  |  |  |  |
| Male | 184 | 34 (18.5%) | 1 |  | 1 |  |
| Female | 428 | 123 (28.7%) | 1.56 (1.11-2.18) | 0.011 | 1.39 (0.98-1.97) | 0.068 |
| **Smoking status** |  |  |  |  |  |  |
| Yes | 29 | 2 (6.9%) | 0.26 (0.07-1.00) | 0.049 | 0.34 (0.09-1.33) | 0.120 |
| No | 583 | 155 (26.6%) | 1 |  | 1 |  |
| **Alcohol use** |  |  |  |  |  |  |
| Yes | 170 | 36 (21.2%) | 0.77 (0.56-1.07) | 0.125 | 0.82 (0.59-1.15) | 0.254 |
| No | 442 | 121 (27.4%) | 1 |  | 1 |  |
| **Physical activity** |  |  |  |  |  |  |
| Moderate/Vigorous | 83 | 16 (19.3%) | 0.72 (0.46-1.15) | 0.170 | 0.82 (0.51-1.32) | 0.422 |
| Low intensity | 529 | 141 (26.7%) | 1 |  |  |  |
| **BMI** | - | - | 1.02 (1.00-1.04) | 0.061 | 1.03 (1.01-1.05) | 0.001 |

ART: antiretroviral therapy; BMI: body mass index; CI: confidence interval; LDL: Low density lipoprotein; PR: prevalence ratio.
