# Supplementary material for: Prevalence of non-communicable diseases among individuals with HIV infection by antiretroviral therapy status in Dar es Salaam, Tanzania
Source: PLoS One. 2020 Jul 9;15(7):e0235542. doi: 10.1371/journal.pone.0235542 (PMC7347196; doi:10.1371/journal.pone.0235542)
Supplement: S1 Appendix — (DOCX) [file pone.0235542.s004.docx]

**S1A Appendix. Interview tool (in English)**

Questionnaire No-------------------

**PART 1: SOCIO-DEMOGRAPHICS**

HEALTH FACILITY …………………. CTC NO………………………...

1. Patient hospital No……………….
2. Age (years)…………….
3. Sex: M/F (circle applicable)
4. Address…………...........
5. Telephone number of patient……………. close relative……………………
6. Marital status
7. Single
8. Married
9. Cohabiting
10. Divorced/Separated
11. Widowed
12. Level of education
13. No formal education
14. Primary education
15. Secondary education
16. Certificate
17. Advanced diploma /Degree
18. Occupation
19. Employed as civil servant
20. Employed in private sector
21. Peasant
22. Self employed
23. Not working

**PART 2: HIV HISTORY**

1. When were you first diagnosed to have HIV (year of diagnosis) --------------
2. Are you on ART? Yes/No - **if no go to Number 14**
3. If yes, for how long (years) --------------------
4. Types (from the records) -----------------------
5. Do you take your ARVs every day? Yes/No
6. Since initiation of ART, were they changed at some point in time? Yes/No
7. If Yes; types, duration of use, reasons for changing

........................... ................................. ..........................

**PART 3: FAMILY AND SOCIAL HISTORY**

1. Are you a smoker currently? Yes/No
2. If Yes, for how long (years) ..........................
3. How many cigarettes per day?.......................
4. If no, were you a smoker in the past? Yes/No
5. If yes, when did you stop smoking?...............
6. Do you drink alcohol? Yes/No
7. If yes, which type?...............................
8. How much per week?..........................
9. Level of physical activities (ascertained from participation in activity categories in the IPAQ questionnaire – short form)
   1. Low intensity (no participation in activities outlined in question 1-4)
   2. Moderate intensity (participation in activities outlined in question 3 & 4 of the IPAQ questionnaire, and no participation in activities outlined in question 1 & 2)
   3. Vigorous intensity (participation in activities outlined in question 1 & 2 of the IPAQ questionnaire)

**PART 4: PAST MEDICAL HISTORY**

1. Are you hypertensive? Yes/No
2. Are you an any antihypertensive? Yes/No
3. Are you diabetic? Yes/No
4. Are you on any treatment for diabetes mellitus? Yes/No
5. Have you ever been told that you have elevated serum lipids? Yes/No
6. Are you on any lipid lowering drugs? Yes/No

**PART 5: EXAMINATION FINDINGS**

Weight (kg) ------------------- Height (m)----------------------- (BMI -------------------------kg/m2)

Blood pressure 1-------------------- 2----------------- 3---------------- (only if the first two differ by >10 mm Hg)

Average (of the last two readings) -------------- (mm Hg)

Fasting plasma blood glucose level------------------------ mmol/L

2HR post prandial glucose level------------------ mmol/L (in an individual with IFG)

Serum creatinine ------------------------------- mmol/L

TC …………. (mmol/L) TGD …………… (mmol/L) HDL ………………… (mmol/L)

LDL ………………. (mmol/L)

**S1B Appendix. International Physical Activity Questionnaire (Short last 7 days self-administered format)**

We are interested in finding out about the kinds of physical activities that people do as part of their everyday lives. The questions will ask you about the time you spent being physically active in the **last 7 days**. Please answer each question even if you do not consider yourself to be an active person. Please think about the activities you do at work, as part of your house and yard work, to get from place to place, and in your spare time for recreation, exercise or sport.

Think about all the **vigorous** activities that you did in the **last 7 days**. **Vigorous** physical activities refer to activities that take hard physical effort and make you breathe much harder than normal. Think only about those physical activities that you did for at least 10 minutes at a time.

1. During the **last 7 days**, on how many days did you do **vigorous** physical activities like heavy lifting, digging, aerobics, or fast bicycling?

_____ **days per week**

No vigorous physical activities **Skip to question 3**

2. How much time did you usually spend doing **vigorous** physical activities on one of those days?

_____ **hours per day**

**_____ minutes per day**

Don’t know/Not sure

Think about all the **moderate** activities that you did in the **last 7 days**. **Moderate** activities refer to activities that take moderate physical effort and make you breathe somewhat harder than normal. Think only about those physical activities that you did for at least 10 minutes at a time.

3. During the **last 7 days**, on how many days did you do **moderate** physical activities like carrying light loads, bicycling at a regular pace, or doubles tennis? Do not include walking.

_____ **days per week**

No moderate physical activities **Skip to question 5**

4. How much time did you usually spend doing **moderate** physical activities on one of those days?

**_____ hours per day**

**_____ minutes per day**

Don’t know/Not sure

Think about the time you spent **walking** in the **last 7 days**. This includes at work and at home, walking to travel from place to place, and any other walking that you have done solely for recreation, sport, exercise, or leisure.

5. During the **last 7 days**, on how many days did you **walk** for at least 10 minutes at a time?

_____ **days per week**

No walking **Skip to question 7**

6. How much time did you usually spend **walking** on one of those days?

**_____ hours per day**

**_____ minutes per day**

Don’t know/Not sure

The last question is about the time you spent **sitting** on weekdays during the **last 7 days**.

Include time spent at work, at home, while doing course work and during leisure time. This may include time spent sitting at a desk, visiting friends, reading, or sitting or lying down to watch television.

7. During the **last 7 days**, how much time did you spend **sitting** on a **week day**?

**_____ hours per day**

**_____ minutes per day**

Don’t know/Not sure

**This is the end of the questionnaire, thank you for participating**.
