# Supplementary material for: Prevalence of non-communicable diseases among individuals with HIV infection by antiretroviral therapy status in Dar es Salaam, Tanzania
Source: PLoS One. 2020 Jul 9;15(7):e0235542. doi: 10.1371/journal.pone.0235542 (PMC7347196; doi:10.1371/journal.pone.0235542)
Supplement: Attachment — Submitted filename: PONE-D-20-00114 18 May 2020.docx [file pone.0235542.s006.docx]

# PONE-D-20-00114

# General comments

The authors have addressed most of the initial suggestions very well. The statistical analysis still needs a little bit more input though:

1. Is there any justification for the statistical approach to modelling used here? In general p-values are not advisable as the criteria for inclusion of variables into models especially in observational studies where p-values could be significant due to chance and/or confounding. Variables that have either biological plausibility and/or evidence of association with the outcome could be included in models despite p-values. In this case, it appears that the variables included have both biological plausibility and literature-based associations with the outcome, so the authors need not change the analysis.
2. The protective effect of smoking reported in this study is, as the authors rightly point out, most likely due to measurement error. Further, the proportion of smokers (29/612 = 5%), and the numbers with outcome (n =6) are very small and may lead to biased estimates of the association. Smoking was not assessed with a validated questionnaire and underreporting is a big concern here. My humble suggestion is for the authors to remove the variable and explain this in either statistical methods or discussion. This may have more benefit than leaving the finding in the table where it may be misinterpreted with the associated potential for harm. I suspect that the model will remain largely unchanged except for the removal of smoking.
