# Supplementary material for: Prevalence of non-communicable diseases among individuals with HIV infection by antiretroviral therapy status in Dar es Salaam, Tanzania
Source: PLoS One. 2020 Jul 9;15(7):e0235542. doi: 10.1371/journal.pone.0235542 (PMC7347196; doi:10.1371/journal.pone.0235542)
Supplement: Attachment — Submitted filename: Response to Reviewers.docx [file pone.0235542.s007.docx]

Dear Editor,

We appreciate the opportunity to revise and resubmit our manuscript titled “Prevalence of non-communicable diseases among individuals with HIV infection by antiretroviral therapy status in Dar es Salaam, Tanzania”. We thank the Editor and reviewers for revision recommendations. We believe the revised manuscript has been strengthened by the recommendations. We provide a response to the reviewer’s comments, a manuscript version with tract changes, and the other without as recommended.

# General comments

The authors have addressed most of the initial suggestions very well. The statistical analysis still needs a little bit more input though:

1. Is there any justification for the statistical approach to modelling used here? In general p-values are not advisable as the criteria for inclusion of variables into models especially in observational studies where p-values could be significant due to chance and/or confounding. Variables that have either biological plausibility and/or evidence of association with the outcome could be included in models despite p-values. In this case, it appears that the variables included have both biological plausibility and literature-based associations with the outcome, so the authors need not change the analysis.

Response:

We note the comments from the reviewer and agree that the analysis remains as presented.

2. The protective effect of smoking reported in this study is, as the authors rightly point out, most likely due to measurement error. Further, the proportion of smokers (29/612 = 5%), and the numbers with outcome (n =6) are very small and may lead to biased estimates of the association. Smoking was not assessed with a validated questionnaire and underreporting is a big concern here. My humble suggestion is for the authors to remove the variable and explain this in either statistical methods or discussion. This may have more benefit than leaving the finding in the table where it may be misinterpreted with the associated potential for harm. I suspect that the model will remain largely unchanged except for the removal of smoking.

Response:

Smoking is a known important factor associated with the development and/or severity of NCDs and should be elicited for in a study on the subject.

We share the views of the reviewer that the apparent ‘protective effect of smoking on NCDs’ as shown in the results section, could be misinterpreted and it is important to prevent this from happening. However, we are uncomfortable with the suggestion that observations on smoking be ‘swept under the carpet’. We believe that we are expected and indeed obliged, to explain the unexpected observation that ‘Smoking is protective to NCDs’. Indeed, we believe most of your readers would want to find out reasons for the observation against the universally accepted norm, which is what we have done in the discussion (interpretation of the finding/result). Consequently, we believe that data on smoking should be presented in the results section. However, having presented our reasoning, we would accept the decision of the editor on the matter.
